# Open for all

**DOI:** 10.1038/bdjopen.2015.4

**Published:** 2015-10-23

**Authors:** Stephen Hancocks

**Affiliations:** 1 Editor-in-Chief, BDJ Open and British Dental Journal

The launch of a new journal is always an exciting moment and it gives me great pleasure to be writing this at the beginning of *BDJ Open*, the new open-access, online-only journal from the British Dental Association (BDA) and Nature Publishing Group.

One of the consequences of online publication has been the advent of universal access to information through the Internet. No longer is access confined to the delivery of paper copies, and what has dawned has been the realisation that research can be shared more widely than ever before. In turn this opens the possibility of making speedier advances in science and medicine by connecting researchers, their work and their findings for the benefit of mankind. However, there are also drawbacks. One fundamental problem is veracity. How can we maintain quality? The peer-review function of journals safeguards this as far as possible, and the answer has been to extend this into the development of open-access journals. Here the premise is that the cost of the publishing process is borne by the author and not by the reader. But this is not ‘vanity’ publishing, at least certainly not as far as reputable journals are concerned, because the author’s work is only accepted and published after the same established process of peer review. It is not a question that merely being prepared to pay gets you published.

Across society we also see a drive for more ‘openness’. In research terms this is reflected by increasing mandates from those funding research for the outputs of research (such as the articles that *BDJ Open* will publish) to be made easily accessible and free to view. *BDJ Open* is designed to address this need, and consequently it is not a subscription journal. Instead, as per the Gold Open Access model, authors of accepted papers *only* (or their institutions) will pay an article-processing charge (APC).

The world of dental research is very wide and using conventional means only the *British Dental Journal* (*BDJ*) is constrained by the amount of content and number of papers it can publish. *BDJ Open* has no such confines and is open, as the name suggests, to research papers across the dental field. To put this in context we currently receive in excess of 900 submissions a year to the *BDJ*, yet we have the capacity to publish only about 150. A quick calculation reveals a ‘rejection’ rate of about 80%, but many of these papers are turned down not because they are substandard but only because they are not appropriate for the *BDJ*’s established content and readership. Our new ability to offer a possible route to publication for many of these papers means that we no longer have to divert them to other journals; we can instead keep them in the *BDJ*’s portfolio of knowledge.

Why should you choose to publish your research in *BDJ Open*?

- *Open access*—all articles are freely available and authors retain copyright. This enables you to reach all clinicians and oral health researchers worldwide, ensuring your article is far-reaching. All *BDJ Open* articles are published under a Creative Commons License.
- *Competitive turnaround times*—Online submission, rapid review and online publication times.
- *Reputation*—The journal is the sister journal of the *BDJ*, which was established in 1881. *BDJ Open* is published in partnership with the BDA and Nature Publishing Group.
- *Visibility*—Content published in *BDJ Open* has the potential to reach dental researchers, medical and dental clinicians, industry and decision-makers globally. Readers have the option to sign up for free table of contents e-alerts, ensuring additional exposure for authors.
- *Article-level metrics*—Using Altmetric.com, readership data and citation information, authors can see how their research is being used and commented on across the scholarly literature, the wider internet and social networks.
- *No format limits*—We have removed the restrictions of print to word limits, colour and figure charges. All articles that conform to the required editorial standards of the journal will be published regardless of length or supplementary content.

We invite you to consider *BDJ Open* when deciding where to submit your next research article. You can do so online at http://mts-bdjopen.nature.com at any time. If you have any questions about this new journal, please contact me via our editorial office at bdjopen@nature.com.

In summary, the launch of *BDJ Open* is an exciting new development for the BDA, our publishing partner, Nature Publishing Group, and, we earnestly believe, for the dental world and outside world in general. With open access fast becoming recognised and validated we feel that this is of great benefit, is best practice in publishing and is a vital next step in the rapid development of a continuing and fascinating journey.

